# Children’s palliative care education and training: developing an education standard framework and audit

**DOI:** 10.1186/s12909-021-02982-4

**Published:** 2021-10-25

**Authors:** Susan Neilson, Duncan Randall, Katrina McNamara, Julia Downing

**Affiliations:** 1 School of Nursing, University of Birmingham, Edgbaston, Birmingham, B15 2TT UK; 2 Faculty of Health and Social Sciences, Department of Nursing Sciences, Bournemouth University, Bournemouth, UK; 3 Association of British Paediatric Nurses, Wolverhampton, UK; 4 International Children’s Palliative Care Network, Bristol, UK

**Keywords:** Children, Paediatric, Palliative care, End of life care, Education, Teaching, Training

## Abstract

**Background:**

The need to align the range of guidance and competencies concerning children’s palliative care and develop an education framework have been recommended by a UK All-Party Parliament Group and others. In response to these recommendations the need for a revised children’s palliative care competency framework was recognized. A Children’s Palliative Care Education and Training Action Group, comprising champions in the field, was formed across UK and Ireland in 2019 to take this work forward. Their aim was to agree core principles of practice in order to standardize children’s palliative care education and training.

**Methods:**

Over four meetings the Action Group reviewed sources of evidence and guidance including palliative care competency documents and UK and Ireland quality and qualification frameworks. Expected levels of developing knowledge and skills were then agreed and identified competencies mapped to each level. The mapping process led to the development of learning outcomes, local indicative programme content and assessment exemplars.

**Results:**

Four sections depicting developing levels of knowledge and skills were identified: Public Health, Universal, Core, Specialist. Each level has four learning outcomes: Communicating effectively, Working with others in and across various settings, Identifying and managing symptoms, Sustaining self-care and supporting the well-being of others. An audit tool template was developed to facilitate quality assurance of programme delivery. The framework and audit tool repository is on the International Children’s Palliative Care Network website for ease of international access.

**Conclusions:**

The framework has received interest at UK, Ireland and International launches. While there are education programmes in children’s palliative care this is the first international attempt to coordinate education, to address lay carer education and to include public health.

## Background

### Children’s palliative care everybody’s business

It is estimated that globally there are around 21 million children in need of palliative and end of life care [1]. There is evidence that this need is increasing for both boys and girls [2]. In addition, the management of palliative care for children has in recent times become a focus of media interest [3] with high profile cases placing healthcare staff under increased pressure. This public attention has been heightened in the Coronavirus pandemic where separation and social distancing has affected palliative care, end of life care and death and grieving rituals for children and their carers as it has for adults [4, 5]. These recent additional pressures could be argued to add to the already challenging emotional and complex cognitive labour of children’s palliative care.

With the growth of the children’s hospice movement across the globe and the coordination of care, education and research through networks such as the International Children’s Palliative Care Network (ICPCN), children’s palliative care is now being considered as an important public health matter. Palliative care across the life span has been gaining more professional and lay attention. In particular, there is now an established community focused on palliative care as a public health issue [6, 7]. These communities include professionals and lay people who work together in the support of the dying and their carers as well as the bereaved. The diversification of care delivery, the integration of hospital, hospice and home care as well as the recognition of a public health focus has led to a need to provide education on children’s palliative care (CPC) not only to health care professionals but, to teams of lay people, informal carers and other professional groups all of whom are involved with healthcare professionals in supporting children and delivering care to children and their carers.

### Rationale for this work

Recommendations for a revised CPC competency framework aligns to guidance suggesting that all professionals have access to a standardised paediatric palliative care curriculum led by facilitators experienced in providing palliative care in both clinical and education and training settings [8–10]. Despite these recommendations [8–11] no educational framework has been established across UK and Ireland.

Children’s palliative care guidance and competencies have been proposed by a number of bodies and organisations, with recommendation for their alignment by the UK All-Party Parliamentary Group on children who need palliative care [8]. This recommendation led to recognition of need for a revised UK competency document [10] and the subsequent formation of the Children’s Palliative Care Education and Training UK and Ireland Action Group in 2019 to take this work forward. Founding members were from Institutes of Higher Education, clinical practice and 3rd sector allied organisations. The aims of the Action Group were to agree core principles of practice and standardise CPC learning.

### International perspective

Whilst it is recognised that education is an essential component of the development of palliative care [12, 13], education on children’s palliative care is not always accessible to those who need it. Although 8 of the more than 21 million children globally needing access to children’s palliative care need specialised care [1], for the vast majority of those children, access to trained health professionals providing palliative care services is not a reality. A systematic review published in 2011 found that 65.6% of countries had no known activities in children’s palliative care with only 5.7% having provision reaching mainstream providers [14]. The situation however has changed since then, and there have been developments in access to palliative care for children in many countries, the pace of change in children’s palliative care development globally and the demand for services means that there is great need for appropriately trained and skilled professionals [15] and the need for education on children’s palliative care has never been greater. Although education systems differ in different countries along with the availability of skilled educators, there is agreement, as reiterated in the World Health Assembly Resolution on Palliative Care [16] for the need for education at different levels: “*Countries should aim to include palliative care as an integral component of the ongoing education and training offered to care providers, in accordance with their roles and responsibilities, according to the following principles:*

- *basic training and continuing education on palliative care*
- *intermediate training for all routinely working with patients with life-threatening illnesses,*
- *specialist palliative care training”(p4)*

Work has been carried out in different regions to identify the competencies required for education on children’s palliative care, such as that by the European Association of Palliative Care White Paper on the core competencies for education in paediatric palliative care [17], which provides recommendations from a multi-disciplinary and multi-national group of CPC professionals and educators. However, the establishment of core standards for curriculum development at the different levels of education provision, is a step forward in achieving an appropriately trained and skilled workforce [18]. Whilst it is essential that any education programme is appropriate for the unique culture, resources, education system of each country, it is also important to acknowledge that globally those working in CPC have a common vision, and that the outcomes of any training on CPC will be improved quality of life for those children and their families with palliative care needs. Thus, whilst adapting and revising programmes and frameworks is essential, it is also important that we do not reinvent the wheel, and can learn from each other in order to increase access to education, as a lack of such education is a challenge to the ongoing development of CPC globally.

### Curriculum design

**Fig. 1:**
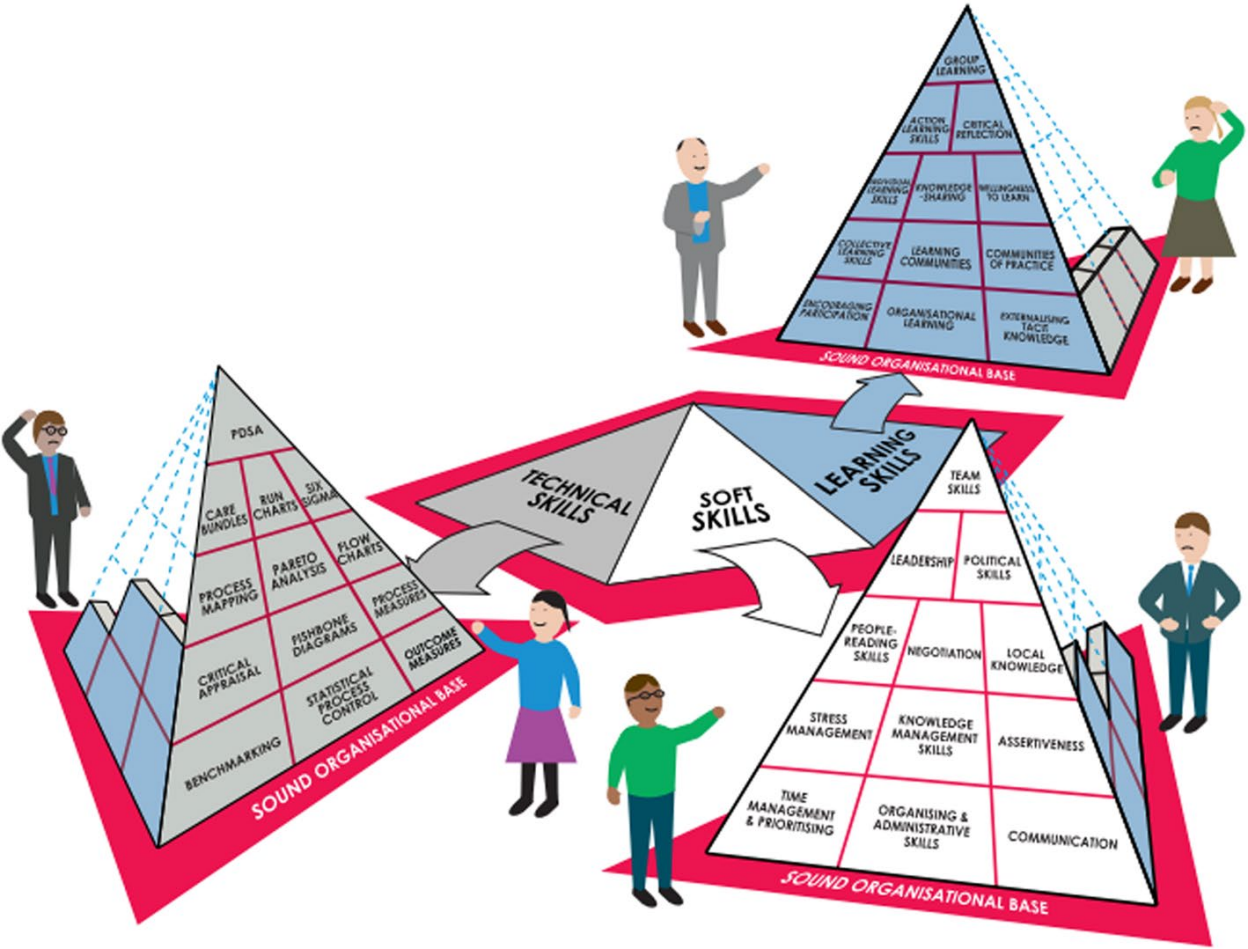
The three-sided improvement pyramid (Gabbay et al. [20]). Reproduced with permission and with credit to the artist Claire Barry

Curriculum design is influenced by local (education provider), intended population and national and international factors. The challenges developing this framework lay in meeting the needs of the range of anticipated interprofessional and lay users and the education providers (Institutes of Higher Education, clinical practice and 3rd sector allied organisations). It is recommended that national qualification frameworks are referred to when designing and approving courses [19]: the developed framework fulfils this need. In addition we wanted to design a framework which would, through education, influence the improvement of children’s palliative care. We decided to use the pyramid approach [20] as this has been found to have good alignment with learning in palliative care teams [21]. The pyramid approach was designed to facilitate service improvements based on best evidence [20]. The approach uses the conception of improvement grounded on a base triangle of organisational support for improvement, and in which teams build improvement in a pyramid fashion learning technical skills, soft skills and learning together skills. Each side of the pyramid being built alongside the others to achieve the health improvement (Fig. 1).

This paper details the process of developing a children’s (0–18 years) palliative care education and training (CPCET) standard framework.

## Methods

**Fig. 2:**
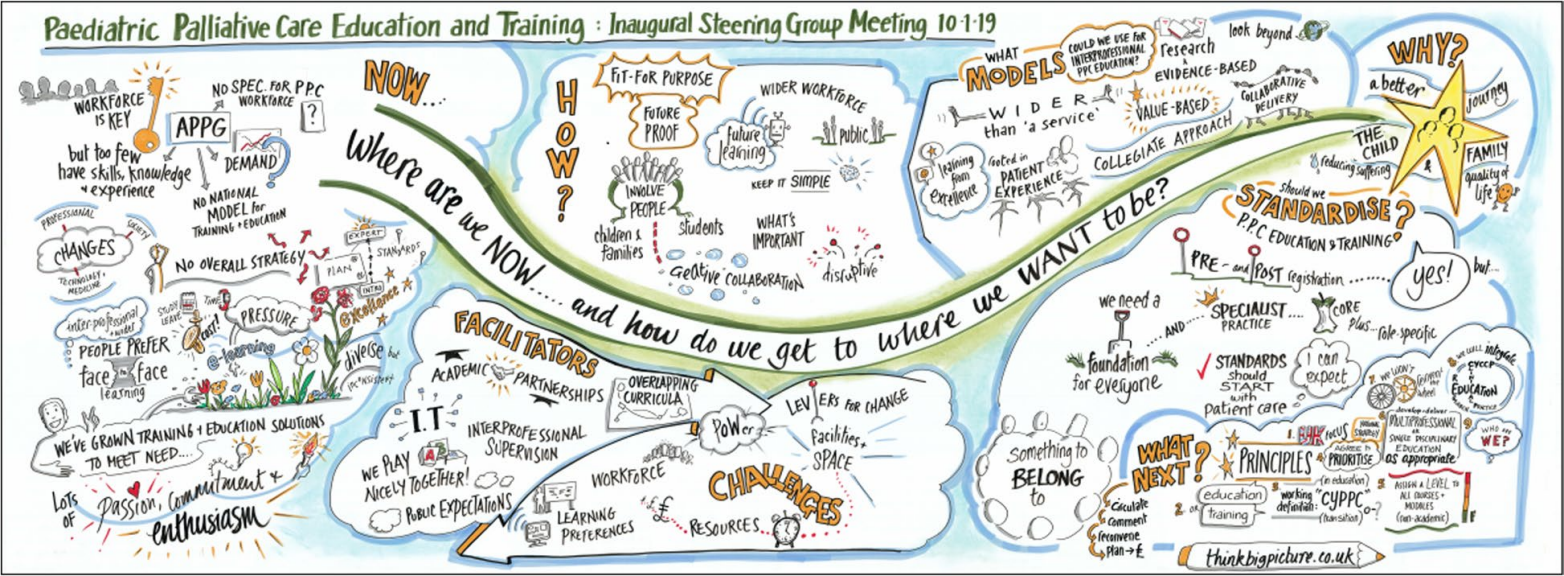
Graphic Illustration of the key themes discussed at the inaugural meeting

On-line searching and word of mouth identified champions from Institutes of Higher Education, clinical practice and 3rd sector allied organisations. Champions were invited to an inaugural meeting to discuss the need to standardise CPC learning. This meeting was attended by 28 delegates. A series of four World Café style [22] focus groups designed by SN and KM explored; the need to standardise CPC education and training in the UK, facilitators and challenges to developing and delivering interprofessional CPC education and training, models for delivery of CPC education and training and how we develop interprofessional education and training that is fit for purpose and ‘future-proof’. Subsequent group feedback and discussion, chaired by Professor Bluebond-Langner (True Colours Chair in Palliative Care for Children and Young People) confirmed that there was a need to standardise CPC learning and agreed core principles of practice. A graphic illustration of the key themes discussed was developed during the meeting (Fig. 2). The UK and Ireland CPCET Action Group was formed with representation from UK and Ireland including the International Children’s Palliative Care Network (ICPCN).

### Programme of work

The plan of work comprised 3 additional meetings. The 35 participants were either members of the inaugural meeting or had subsequently joined the working group through word of mouth. All were from either Institutes of Higher Education, clinical practice or 3rd sector allied organisation. Participants were not paid for their attendance at meetings or for work undertaken during or outside of the meetings. In the first meeting a CPC definition was agreed along with the working group name and terms of reference. Three work streams identified in the inaugural meeting (i) Standardisation of programmes to include an education framework and related career pathways (nursing), ii) Wider participation iii) Funding, were discussed and revised. The agreed working groups were i) levels of interprofessional education and training (lead DR), National career pathways (Lead KM), Foundation programme (Lead Lizzie Chambers) and Education framework (SN). Group members were assigned according to preference. Collated information was fed back at the subsequent meeting. The aim of the second meeting was to develop and then populate the Framework template with information from the group work. During this process consideration was given to underpinning evidence such as the course design pyramid approach [20], and Blooms Taxonomy [23]. Recommended for designing courses the Gabby et al., pyramid approach [20], suggests that technical and soft skills (values and attitudes) need to be combined with learning as a team in order to improve practice. Blooms Taxonomy’s hierarchical classification [23] was used to inform the development of the learning outcomes. The education standard framework learning outcomes were drawn from four dimensions of a competency framework: ‘*Communicating effectively with children and young people with palliative care needs; Providing multidisciplinary holistic care to children and young people with palliative care needs in any care setting (hospital, hospice, the home, school or other community setting); Working with primary carers and health care professionals to identify and manage symptoms; Sustaining self and the wellbeing of others when caring and supporting children/young people and families with their grief, loss and bereavement’* [24]. From each of these core statements a series of learning outcomes were developed. The aim of the final meeting was to review the mapping and sense check the documents. Inclusion of suggested local indicative content examples were recommended during this meeting and developed following the meeting by SN and DR. A final wider review of the documentation was then undertaken by email.

## Results

The depicted process details the steps taken in developing a CPC education and training standard framework. The framework details levels of knowledge, skills and performance that are considered to help health care workers deliver safe, effective and achievable practice. It provides guidance on course content to educationalists; ensuring a standardised curriculum content but with flexibility of delivery and assessment. Development of the Framework involved systematically identifying and then mapping information around assessment credit and quality, CPC competency documents and training programmes. Examples used to inform the Universal Framework level are shown in Table 1.

**Table 1:** Examples of resources used to inform the Universal Framework level

Quality and Qualifications Ireland.	
Scottish Credit and Qualifications Framework.	
The Quality Assurance Agency for Higher Education.	
Health Education England End of Life Care Core Skills Education and Training Framework.	
ICPCN eLearning programme.	
Palliative and End of Life Care: A framework to support the learning and development needs of the health and social service workforce in Scotland.	

Four sections depicting the expected levels of developing knowledge and skills: Public Health, Universal, Core and Specialist were identified (Table 2).

**Table 2:** Framework Levels

Level	Description
Public Health	In this level children’s palliative care as a public health issue will be addressed. Aspects such as social attitude to death and dying in childhood and bereavement following a child death are explored. This would be expected to be across education, health and social care and involve other stakeholder groups concerned with children, their experience of childhood, learning and support of children, siblings, parents and other family members as well as communities affected by child death (e.g. school communities).
Universal	In this level the needs will be addressed of all people working in institutions or facilities which provide care and support to children and their carers. It addresses what any person working in such environments is likely to need to understand about children’s palliative care. This includes clinical and non-clinical staff. Where children’s palliative care is everyone in the workplaces business.
Core	In this level the focus will be on the learning for people who deliver care to children and their carers. It includes everyone who delivers care to children in education, social and health care who might encounter a child living with a life limiting/threatening condition and or the child’s carers (family and communities). The core programmes for sectors of health, education and social care might be different to address the needs of children accessing these types of care. In healthcare this level should include care of the dying child and their carers as well as supporting people with loss and bereavement following a child’s death.
Specialist	In this level leadership and management of palliative and end of life care for children is the focus. It includes clinical, research, education and management leadership. As well as addressing the needs of children and carers with complex and or multiple palliative care needs it would prepare practitioners to be a resource for those learning and delivering care at the other levels. This level includes learning to deliver end of life care in complex situations or where symptom management is challenging.

The four levels, designed to be accessed by people from different professional background, or no professional background, can be viewed as standalone or incremental steps. The number of levels required would be determined by a person’s role and work, for example a specialist children’s palliative care practitioner may require all 4 levels. Children’s palliative care as a public health issue is addressed in the Public Health level. The Universal level addresses the needs of people working in settings where palliative care is provided to children and their families. The Core level provides education standards for use by all professional groups working directly with children with palliative and end of life care needs. This professional group would regularly deliver direct care to these children and their families but may also work with children with other health, social and educational needs. The final level, Specialist, focuses on leadership and management of children’s palliative care encompassing clinical, research, education and management leadership.

The intended learning outcomes were informed by collated competencies and frameworks such as a UK competency framework developed for all health care professionals involved in the provision of palliative care to infants, children and young people [23], Core Competencies for Education in Children’s Palliative Care [10] and the Combined Curriculum in Paediatric Palliative Medicine [25]. The learning outcomes describe the knowledge and skills that should be achieved on completion of a programme of learning (Table 3) ensuring a standardised approach.

**Table 3:** Extract from the Core level of the Framework

Level	Learning outcomes	Suggested local indicative content exemplars	Suggested assessment exemplars
**Diploma** QAA England Wales and N Ireland (FHEQ): 4–5 Scotland SCQF: 8–9 Ireland QQI: 6–9	**2. Working with others in and across various settings.** Analyse the practice and approaches to identifying palliative and end of life care needs of children and their carers. Identify and reflect on your own role within the team delivering palliative and end of life care. Discuss professional roles and responsibilities in a multi-disciplinary (or inter-professional) team delivering children’s palliative and end of life care. Explain the legal and practical requirements related to the care of a child’s body after death.	**2. Working with others in and across various settings, for example:** Children and carer’s palliative and end of life care needs. Positive discussion of needs with children and their carers. Legal and ethical issues in palliate care. Understanding advocacy for children and for carers in palliative care contexts. Working with other professional groups in own organisation and in other settings. Team dynamics and own role in delivering palliative care. Policies and procedures to deal with a child death including care of the body after death. Legal aspects of caring for a child during and following death in country/state/territory.	Assignment: word count 2000–3000 words. On-line MCQ with feedback.

Each learning outcome develops incrementally across the 4 levels. For example, at Public Health level the expectation around communication for learners would be to: *‘Gain an appreciation of both ‘helpful’ and ‘unhelpful’ patterns of communication with children and their carers who are living with life-limiting/life threatening conditions and those who have experienced a bereavement.’* This contrasts with expectation at Specialist level: *‘Analyse cultures and patterns of communication in managing complex issues and in children’s palliative and end of life care.’*

The suggested examples columns were included to help educationalists constructively align their programmes of learning. Once the learning outcomes had been agreed, suggested assessment exemplars were written (Table 3) ensuring constructive alignment of teaching and learning and assessment criteria to the identified learning outcomes [26].

An audit tool (Table 4) was then developed for each of the 4 framework levels primarily to enable education leads to review their course/module. The tool details how the course/module was aligned to the learning outcomes, methods of assessment and any changes undertaken since it was last run. The tool also contains a section for student feedback and a development action plan. There are sections to review each learning outcome in turn: educationalists can reflect on the delivered content (what was taught and how it was taught) and how it was assessed (for example a written or on-line multiple-choice exam or practical exam, such as an objective structured clinical examination). The third column is for recording any revisions that were made to the course delivery. The development action plan details developments required, anticipated outcome(s) from improvements, actions to be undertaken to achieve these and the lead responsible for taking this work forward.

**Table 4:** Audit tool extract

Children’s Palliative Care Education and Training United kingdom and Ireland Education Standard Framework Audit Tool					
**Module name**					
**Organisation name**					
**Course/module lead**					
**Email contact**					
**Date of audit**					
**Signature(s)**					
**AUDIT**					
Learning outcome	Aligned course content		Method(s) of assessment	Changes made during last audit	
**STUDENT FEEDBACK ON COURSE/MODULE**					
**DEVELOPMENT ACTION PLAN**					
Learning outcome identified for development	Audit improvement outcome	Actions	Lead name	Date	Outcome Achieved Partially achieved Not achieved

A final mapping review and sense check of all the documents was undertaken by SN and DR.

## Discussion

We believe this is the first attempt to coordinate education programmes not just across different aspects of children’s palliative care, but across disciplines and professional groups as well as including lay carers working in palliative care organisations. Guidance and templates can be downloaded and completed audits uploaded to the ICPCN website [27].

When considering curriculum design, the pyramid approach [20] views technical, soft skills and learning as sides of a pyramid with simultaneous development of each side of the pyramid required to build the pyramid. It is recognized that development of the pyramid, and learning, however, will be hindered by over emphasis of one aspect. Gabby and her colleagues note the importance of a secure foundation for the pyramid, in this context a comprehensive institutional commitment to children’s palliative care underpinned by well-developed understanding and policy is required. Technical skills (for example, the learning outcome *Identifying and managing symptoms*), soft skills (for example, the learning outcome *Sustaining self and well-being of others*) and learning together (for example, the learning outcome *Communicating effectively*) should therefore be included in programmes of learning. The overall premise is that effective learning will improve the quality of care provided. Constructive alignment within curriculum design (alignment of teaching and learning and assessment criteria to the identified learning outcomes [26]) helps facilitate targeted learning through the use of appropriate teaching and learning activities that meet the intended learning outcomes of individual courses. The rationale for this is that a constructively aligned programme of learning helps facilitate a supportive learning environment and thereby aids learning.

The framework complements and develops highlighted previous international work undertaken [17] identifying competencies required for CPC. In detailing levels of learning, learning outcomes, local indicative programme content and assessment exemplars the framework helps inform a standardised approach to CPC learning across Public Health to Specialist levels. This non prescriptive approach affords the educationalist flexibility and control in their curriculum development and delivery and has global applicability.

Educationalists also are responsible for verification that participants accessing standalone levels above the Public Health level have met learning outcomes of prior levels. Providers of CPC education and training can map their modules to the framework thereby ensure a standardised approach. On completion of the course educators can complete the audit to identify areas for development with the aim of enhancing the teaching-learning process. However, whether the self-verification approach is effective in monitoring and improving the quality of education programmes remains to be determined. In addition, we are not aware of any literature which evaluates the use of the four levels, including the integration of professional and lay carers groups, nor if such an approach leads to substantial improvements in the delivery of children’s palliative care.

We recognise a limitation to this work. The profile of the attendees was not collected thus we cannot attest to the representation of various stakeholders nor of points of view. There may be groups not included in our meetings, however, the invitation was shared widely and a diverse group of people from many relevant stakeholders were present.

### Future work

Translation of the framework and audit is being actively explored in order to promote international accessibility. In addition, the development of training workshops and a buddy system for support and sharing of knowledge and expertise are being considered. An audit repository on the ICPCN website provides opportunity for a planned review of completed audits to inform future children’s palliative care education and training.

## Conclusions

The framework and audit has been developed to help educationalists coordinate and quality assure their programmes of learning. This work commenced pre-Covid19 pandemic and was completed and launched mid-pandemic (the UK and Ireland launch November 2020 and international launch February 2021), highlighting the importance of the work to those involved.

The framework provides a means of standardising children’s palliative care knowledge and competencies across 4 incremental levels. Its novelty lies in the inclusion of a public health level, the applicability to all professional and lay groups working in children’s palliative care, and having an associated audit tool to enable educationalists to quality assure their programmes of learning.

## Data Availability

Not applicable.

## References

[1] Connor SR, Downing J, Marston J (2017). Estimating the global need for palliative care for children: a cross-sectional analysis. J Pain Symptom Manag.

[2] Fraser L (2012). Rising national prevalence of life-limiting conditions in children in England. Pediatrics.

[3] Shah SK, Rosenberg AR, Diekema DS (2017). Charlie Gard and the limits of best interests. JAMA Pediatr.

[4] Breen LJ, Lee SA, Neimeyer RA (2021). Psychological risk factors of functional impairment after COVID-19 deaths. J Pain Symptom Manag.

[5] aTTitude Technical Scientific council. IPSS Covid 19 and pediatric palliative care [video], 2020. https://www.youtube.com/watch?v=Wj7qnTFCkNE&feature=emb_logo

[6] Kellehear A (2013). Compassionate communities: end of life care as everyone’s responsibility. QJM.

[7] Sallnow L, Kumar S, Kellehear A (2012). International Perspectives in Public Health and Palliative Care.

[8] Cooper J. End of life care: strengthening choice. An inquiry report by the All-Party Parliamentary Group (APPG) for Children Who Need Palliative Care, 2018. https://www.togetherforshortlives.org.uk/wpcontent/uploads/2018/10/Pol_Res_181019_APPG_Children_Who_Need_Palliative_Care_inquiry_report.pdf

[9] Department of Health (2016). Providing high quality palliative care for our children. A strategy for children’s palliative and end of life care 2016–26.

[10] Downing J, Ling J, Benini F, Payne S, Papadatou D (2013). Core competencies for education in Paediatric palliative care.

[11] Steering Committee of the European Association for Palliative Care. European Association for Palliative Care IMPaCCT: standards for paediatric palliative care in Europe. Eur J Palliat Care. 2007;14(3):109–14.

[12] European Association for Palliative Care (EAPC), De Vlieger, M, Gorchs, N, et al. A guide for the development of palliative nurse education in Europe. Milano: EAPC Onlus; 2004.

[13] Stjernsward J, Foley KM, Ferris FD (2007). The public health strategy for palliative care. J Pain Symptom Manag.

[14] Knapp C, Woodworth L, Wright M, Downing J, Drake R, Fowler-Kerry S, Hain R, Marston J (2011). Pediatric palliative care provision around the world: a systematic review. Pediatr Blood Cancer.

[15] Ferguson L, Fowler-Kerry S, Hain RDW, Goldman A, Hain R, Liben S (2012). Education. Oxford Textbook of Palliative Care for Children.

[16] World Health Organization (2014). Strengthening of palliative care as a component of integrated treatment throughout the life course. J Pain Palliat Care Pharmacother.

[17] Downing J, Ling J, Benini F, Payne S, Papadatou D (2014). A summary of the EAPC white paper on core competencies for education in paediatric palliative care. Eur J Palliat Care.

[18] Downing J (2012). Ling J education in children’s palliative care across Europe and internationally. Int J Palliat Nurs.

[19] The Quality Assurance Agency for Higher Education (2018). UK Quality Code for Higher Education Advice and Guidance Course Design and Development.

[20] Gabbay J, Le May A, Connell C, Klein JH (2014). Skilled for improvement? Learning communities and the skills needed to improve care: an evaluative service development.

[21] The World Café Community Foundation The Word Café, 2021. http://www.theworldcafe.com/

[22] Randall D, Garbutt D, Barnard M (2018). Using simulation as a learning experience in clinical teams to learn about palliative and end-of-life care: a literature review. Death Studies.

[23] Bloom BS (1956). Taxonomy of educational objectives: the classification of educational goals.

[24] Royal College of Nursing RCN competencies: Caring for Infants, Children and Young People requiring Palliative Care [page 8], 2018. https://www.rcn.org.uk/professional-development/publications/pub-007033

[25] Association for Paediatric Palliative Medicine Combined Curriculum in Paediatric Palliative Medicine [on-line], 2015. https://www.appm.org.uk/training/combined-curriculum/

[26] Biggs J (1996). Enhancing teaching through constructive alignment. High Educ.

[27] UK and Ireland Children’s Palliative Care Education and Training Action Group (2020). Children’s Palliative Care Education Standard Framework.

